# 87 Intracerebral inflammatory pseudotumor in Behçet’s disease: a pediatric case report

**DOI:** 10.1093/rheumatology/keac496.083

**Published:** 2022-10-07

**Authors:** Djohra Hadef, Samy Slimani, Mouna Nezzar, Issam Tahrour

**Affiliations:** 1 Department of Pediatrics, University Hospital Center of Batna, Faculty of Medicine, Batna 2 University; 2 Atlas Clinic of Rheumatology, Batna, Algeria; 3 Pediatric Oncologist, CAC Batna, Algeria; 4 Private Clinic of Rheumatology, Batna, Algeria

## Abstract

**Introduction:**

Behçet's disease is a chronic, relapsing, multisystem vasculitis. The diagnosis is essentially clinical, due to the absence of specific biological criteria, which remains very difficult to establish in pediatric age because of often insidious or atypical disease onset. The association of Pseudo Inflammatory Non-Specific Tumors (PTINS) and Behcet's disease (BD) is exceptionally reported. We report the case of a 12-year-old boy with Behçet's disease who presented with an inflammatory pseudotumor during the course of his disease.

**Methods and results:**

A 12-year-old boy, from Batna (Algeria), with no relevant past medical history, admitted in 2010 for the management of a venous thrombosis of the right lower limb without any obvious cause and which responded well to anticoagulant treatment.

A year later, the child was readmitted for polymorphic clinical signs made up of joint damage (inflammatory arthralgia of the large joints), digestive (glairo-bloody diarrhea) and ocular (decreased visual acuity) and skin and mucous membranes (oral-genital apthosis and erythema nodosum of the 4 limbs).

Lab work-up showed significant inflammatory syndrome with erythrocyte sedimentation rate (ESR) of 100 mm at the first h, C-reactive protein (CRP) up to 400 mg/l and thrombocytosis (700 000–900 000 elements/mm2). HLA B51 was negative.

The patient was treated with Colchicine, Steroids, and Aspirin with partial response. Subsequently, a treatment with azathioprine was started, with clinical improvement (despite occasional digestive crises) but with persistent biologic inflammation.

In 2012 the patient presented with a unilateral decrease in visual acuity. A CT scan was performed showing an intracranial tumor compressing the right optic nerve with all the criteria of a malignant tumor (rupture of the cortex, increase in volume on 2 successive CT scans). A transsphenoidal biopsy of the mass revealed a non-specific inflammation. Biological treatment with anti-TNF alpha (Infliximab) was associated with a spectacular tumor regression, allowing retrospectively the diagnosis of an inflammatory granulomatous lesion related to Behçet's disease rather than a malignant tumor. The outcomes were favorable with clinical and biological improvement for the first time since the onset of the disease.

**Conclusion:**

Behcet is rare in the pediatric age group and it is difficult to diagnose. Nonspecific inflammatory pseudotumors can be associated with Behçet's disease and constitute a real diagnostic and therapeutic challenge.

**Disclosure of Interest:**

None declared

